# Annual report of the Japanese Breast Cancer Society registry for 2016

**DOI:** 10.1007/s12282-020-01081-4

**Published:** 2020-05-11

**Authors:** Makoto Kubo, Hiraku Kumamaru, Urara Isozumi, Minoru Miyashita, Masayuki Nagahashi, Takayuki Kadoya, Yasuyuki Kojima, Kenjiro Aogi, Naoki Hayashi, Kenji Tamura, Sota Asaga, Naoki Niikura, Etsuyo Ogo, Kotaro Iijima, Kenta Tanakura, Masayuki Yoshida, Hiroaki Miyata, Yutaka Yamamoto, Shigeru Imoto, Hiromitsu Jinno

**Affiliations:** 1 Department of Surgery and Oncology, Graduate School of Medical Sciences, Kyushu University, 3-1-1 Maidashi Higashi-ku, Fukuoka, 812-8582 Japan; 2 Department of Healthcare Quality Assessment, University of Tokyo, 7-3-1 Hongo, Bunkyo-ku, Tokyo, 113-8655 Japan; 3 Department of Breast and Endocrine Surgical Oncology, Tohoku University School of Medicine, Seiryo-machi, Aoba-ku, Sendai, 980-8574 Japan; 4 Division of Digestive and General Surgery, Niigata University Graduate School of Medical and Dental Sciences, 1-757 Asahimachi-dori, Chuo-ku, Niigata, 951-8510 Japan; 5 Department of Surgical Oncology, Research Institute for Radiation Biology and Medicine, Hiroshima University, 1-2-3 Kasumi, Minami-ku, Hiroshima, 734-0037 Japan; 6 Division of Breast and Endocrine Surgery, Department of Surgery, St. Marianna University School of Medicine, 2-16-1 Sugao, Miyamae-ku, Kawasaki, 216-8511 Japan; 7 Department of Breast Oncology, National Hospital Organization Shikoku Cancer Center, Kou 160, Minamiumemotomachi, Matsuyama, Ehime 791-0280 Japan; 8 Department of Breast Surgical Oncology, St. Luke’s International Hospital, 9-1 Akashicho, Chuo-ku, Tokyo, 104-8560 Japan; 9 Department of Breast and Medical Oncology, National Cancer Center Hospital, 5-1-1 Tsukiji, Chuo-ku, Tokyo, 104-0045 Japan; 10 Department of Breast Surgery, Kyorin University Hospital, 6-20-2 Shinkawa, Mitaka, Tokyo 181-8611 Japan; 11 Department of Breast and Endocrine Surgery, Tokai University School of Medicine, 143 Shimokasuya, Isehara, Kanagawa 259-1193 Japan; 12 Department of Radiology, Kurume University School of Medicine, 67 Asahi-Machi, Kurume, Fukuoka 830-0011 Japan; 13 Department of Breast Oncology, Juntendo University, 3-1-3 Hongo, Bunkyo-ku, Tokyo, 113-8431 Japan; 14 Department of Plastic and Reconstructive Surgery, Mitsui Memorial Hospital, Kanda-Izumi-cho 1, Chiyoda-ku, Tokyo, 101-8643 Japan; 15 Department of Diagnostic Pathology, National Cancer Center Hospital, 5-1-1 Tsukiji, Chuo-ku, Tokyo, 104-0045 Japan; 16 Department of Molecular-Targeting Therapy for Breast Cancer, Kumamoto University, 1-1-1 Honjo, Chuo-ku, Kumamoto, 860-8556 Japan; 17 Department of Surgery, Teikyo University School of Medicine, 2-11-1 Kaga, Itabashi-ku, Tokyo, 173-8606 Japan

**Keywords:** Japanese Breast Cancer Society, Breast Cancer Registry, National Clinical Database, Menstruation, Nodal status

## Abstract

The Japanese Breast Cancer Society (JBCS) registry began data collection in 1975, and it was integrated into National Clinical Database in 2012. As of 2016, the JBCS registry contains records of 656,896 breast cancer patients from more than 1400 hospitals throughout Japan. In the 2016 registration, the number of institutes involved was 1422, and the total number of patients was 95,870. We herein present the summary of the annual data of the JBCS registry collected in 2016. We analyzed the demographic and clinicopathologic characteristics of registered breast cancer patients from various angles. Especially, we examined the registrations on family history, menstruation, onset age, body mass index according to age, nodal status based on tumor size and subtype, and proportion based on ER, PgR, and HER2 status. This report based on the JBCS registry would support clinical management for breast cancer patients and clinical study in the near future.

## Preface

The Japanese Breast Cancer Society (JBCS) registry began data collection in 1975, and started a new web-based system with the cooperation of the non-profit organization, Japan Clinical Research Support Unit and the Public Health Research Foundation (Tokyo, Japan) in 2004. The registry, starting in 2012, runs on the National Clinical Database (NCD) which is a multidisciplinary registry platform for interventional and cancer registries. The details were described previously [1]. The eligibility for registration is that patients were diagnosed to have a new onset breast cancer at NCD participating facilities throughout Japan. The registration criteria do not require the patient to have undergone a breast surgery. As NCD does not support the linkage of a patient across hospitals, double registration may occur especially for the cases without breast surgery. However, as 97.4% of patients registered in 2016 had breast surgery, there are few cases with double registration. As of 2016, it contains records of 656,896 breast cancer patients from more than 1400 hospitals throughout Japan. Affiliated institutions provide data covering more than 50 demographic and clinicopathologic characteristics of newly diagnosed primary breast cancer patients via a web-based registration system. Follow-up information on 5-, 10-, and 15-year prognosis after the first treatment (preoperative systemic therapy or surgery) is requested. The JBCS registry is directed and governed by the Registration Committee of JBCS. TNM classification is now registered according to the 7th edition of the Union for International Cancer Control staging system [2], and histological classification is registered according to the General Rules for Clinical and Pathological Recording of Breast Cancer [3], which was further transferred to the Classification of Tumors of the Breast and Female Genital Organs [4].

**Fig. 1:**
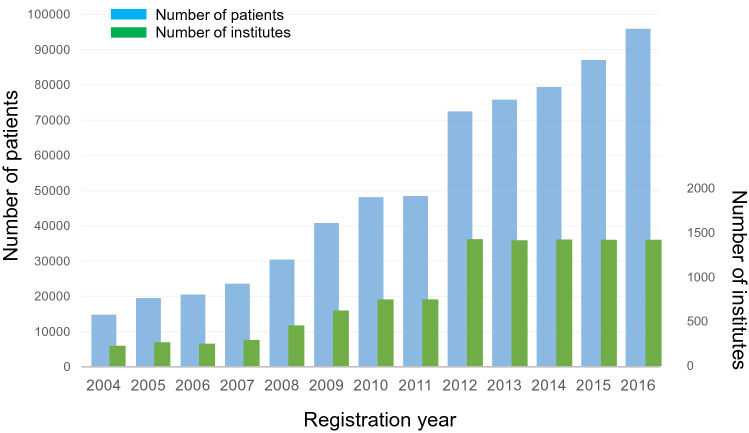
Changes in the number of patients and institutes over time

**Fig. 2:**
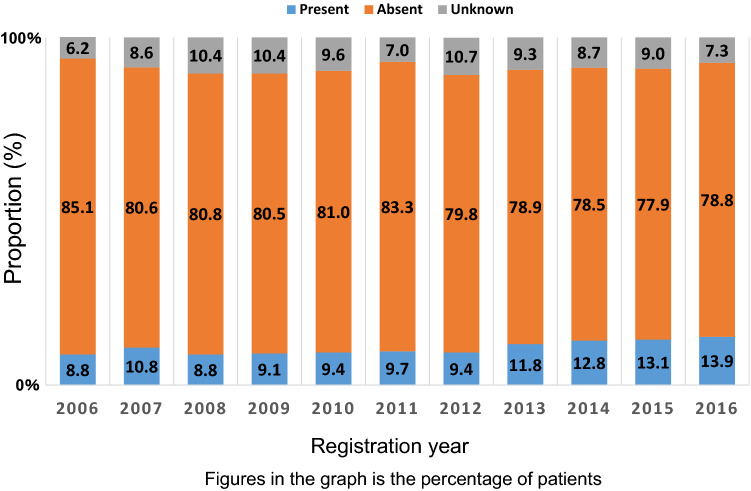
Frequencies of the patients with a family history based on patient interviews

**Fig. 3:**
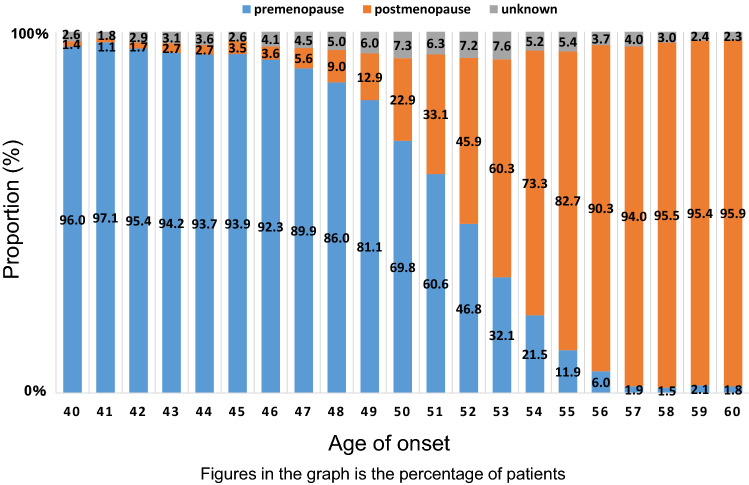
Menopausal status

**Fig. 4:**
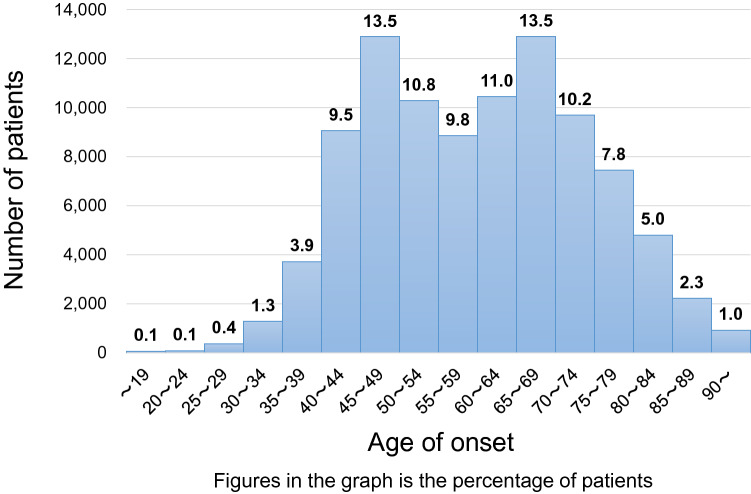
Distribution of onset age

**Fig. 5:**
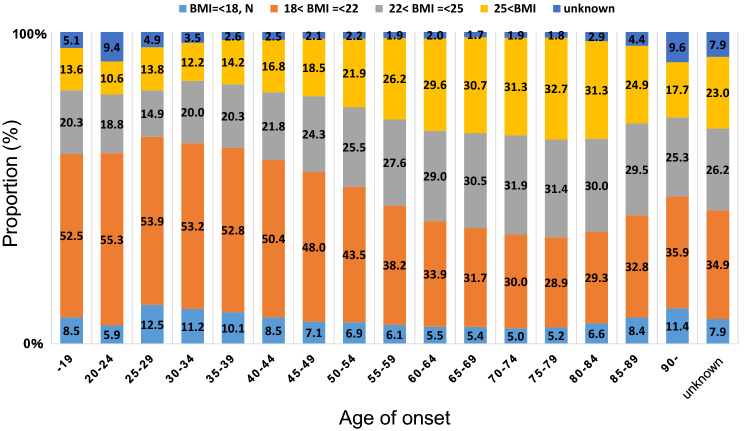
Body mass index (BMI) according to age

**Fig. 6:**
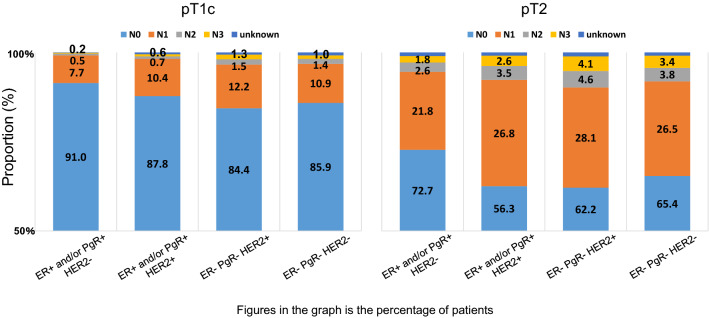
Nodal status based on tumor size and subtype

**Fig. 7:**
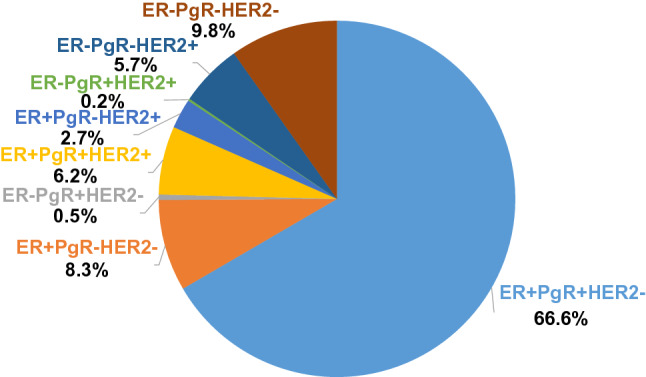
Proportion based on ER, PgR, and HER2 status

Herein, we present the summary of the annual data of JBCS registry collected in 2016 (Tables 1, 2, 3; Figs. 1, 2, 3, 4, 5, 6, 7). The number of institutes involved in the 2016 registration was 1422, and the total number of patients was 95,870, including 5803 patients with simultaneous bilateral breast cancers. The incidence per year of breast cancer, including ductal carcinoma in situ, was reported to be 107,627 in 2016 by the National Cancer Center and the Ministry of Health, Labor and Welfare [5, 6]. Thus, approximately 84% of newly diagnosed breast cancer patients were included in the JBCS registry in 2016. While the number of patients has increased, the number of institutes has not increased since NCD was started in 2012 (Fig. 1). As a result, the number of registered patients per institute has gradually increased.

**Table 1:** Patients' characteristics

All	*N* = 95,870	%
Gender		
Female	95,257	99.4
Male	613	0.6

Female	*N* = 95,257	%
Unilateral	85,973	90.3
Bilateral		
Synchronous	5803	6.1
Metachronous	3479	3.7
Family history		
Absent	75,073	78.8
Present	13,197	13.9
Unknown	6985	7.3
Menstruation		
Premenopausal	31,255	32.8
Postmenopausal	61,252	64.3
Unknown	2748	2.9
Surgery		
Present	91,541	96.1
Absent	662	0.7
Biopsy alone	3054	3.2
Tumor size		
Tis	13,069	13.7
T0	444	0.5
T1	44,905	47.1
T2	27,636	29.0
T3	2933	3.1
T4	4609	4.8
Unknown	1661	1.7
Nodal status		
N0	77,035	80.9
N1	12,700	13.3
N2	2009	2.1
N3	1735	1.8
Unknown	1778	1.9
Metastasis		
M0	91,362	95.9
M1	1957	2.1
Unknown	1938	2.0
Stage		
0	12,986	13.6
I	41,490	43.6
IIA	22,134	23.2
IIB	7655	8.0
IIIA	2200	2.3
IIIB	3098	3.3
IIIC	1229	1.3
IV	1957	2.1
Unknown	2508	2.6

TNM classifications were identified using the UICC staging system

The TNM classifications in this Table are from clinical data

**Table 2:** Comparison of clinical and pathological classifications

			pTis		pT1		pT2		pT3		Unknown	
	*n*	%	*n*	%	*n*	%	*n*	%	*n*	%	*n*	%
(a) Tumor size												
cTis	12,618	16.4	4963	39.3	3805	30.2	1356	10.7	511	4.0	1983	15.7
cT0	383	0.5	66	17.2	148	38.6	39	10.2	4	1.0	126	32.9
cT1	40,446	52.6	1276	3.2	32,178	79.6	4181	10.3	453	1.1	2358	5.8
cT2	20,007	26.0	267	1.3	5050	25.2	12,583	62.9	898	4.5	1209	6.0
cT3	1494	1.9	18	1.2	111	7.4	474	31.7	770	51.5	121	8.1
cT4	1563	2.0	7	0.4	179	11.5	799	51.1	421	26.9	157	10.0
Unknown	354	0.5	19	5.4	91	25.7	44	12.4	33	9.3	167	47.2
Total	76,865	100.0	6616	8.6	41,562	54.1	19,476	25.3	3090	4.0	6121	8.0

Node	Clinical		Pathological		
	*n*	%	N+	*n*	%
(b) Nodal status					
Negative	68,872	89.6	0	52,126	75.7
			1–3	7235	10.5
			4–9	842	1.2
			10≤	273	0.4
			Unknown	8396	12.2
Positive	7730	10.1	0	822	10.6
			1–3	3849	49.8
			4–9	1467	19.0
			10≤	915	11.8
			Unknown	677	8.8
Unknown	263	0.3	Unknown	263	
Total	76,865	100.0	Total	76,865	

The TNM classification was identified by the UICC staging system

*N+* number of involved nodes

**Table 3:** Differences of biological features distinguishing distant metastasis (M0 and M1)

		M0		M1	
		*N* = 91,362	%	*N* = 1957	%
ER					
Negative		12,967	14.2	424	21.7
Positive	1–9%	2898	3.2	72	3.8
	≥ 10%	65,922	72.1	1193	61.0
NE		6545	7.2	146	7.5
Unknown		3030	3.3	122	6.2
PgR					
Negative		21,202	23.2	704	36.0
Positive	1–9%	6744	7.4	185	9.5
	≥ 10%	53,577	58.6	798	40.8
NE		6769	7.4	148	7.6
Unknown		3070	3.4	122	1.7
HER2					
Negative		62,101	68.0	1185	60.5
Positive		10,674	11.7	386	19.7
NE		12,060	13.2	201	10.3
Unknown		6527	7.1	185	9.5
HER2/IHC					
0		26,984	29.5	533	27.2
1+		27,334	29.9	485	24.8
2+	Equivocal	12,892	14.1	299	15.3
2+/ISH	Positive	1957	15.2	69	23.1
	Negative	7783	60.4	167	55.8
	NE	2831	21.9	60	20.1
	Unknown	321	2.5	3	1.0
3+		8717	9.5	317	16.2
NE		12,060	13.2	201	10.3
Unknown		3375	3.7	122	6.2

		M0		M1	
		*N* = 88,819	%	*N* = 1957	%
Nuclear grade					
1		32,699	36.8	238	18.6
2		25,445	28.6	334	26.2
3		15,514	17.5	371	29.1
NE		7814	8.8	115	9.0
Unknown		7347	8.3	219	17.1

*ER* estrogen receptor, *PgR* progesterone receptor, *HER2* human epidermal growth factor receptor 2, *IHC* immunohistochemistry, *ISH* in situ hybridization, *NE* not examined

The TNM classification was identified by the UICC staging system

## Summary of findings

Among the 95,870 patients, 95,257 were women (99.4%) and the mean ± standard deviation of onset age was 59.7 ± 13.9 years. We show data of patient characteristics on female breast cancer, such as unilateral or bilateral disease, family history, menstruation, operation, tumor size, nodal status, metastasis, and stage in Table 1. There were 13,197 (13.9%) patients with a family history of breast cancer. Family history in NCD means that at least one first- or second-degree relative have a history of breast cancer. Patients with family history of breast cancer based on patient interviews have increased since 2013, perhaps reflecting our growing interest in the family history of hereditary tumors around that time (Fig. 2). This is also supported by the decreasing proportion of those with “unknown” family history status. According to the meta-analysis in United Kingdom, it was reported that at least one first-degree relative had a history of breast cancer in 12.9% of breast cancer patients [7], which is similar to the proportion in this report, but the true reason of the increased proportion of patients with a family history of breast cancer is unclear in this study.

Moreover, we found that 33% of breast cancer patients were premenopausal (Table 1), which is closely related to the distribution of onset age. To view this from another angle, we analyzed data on menstruation by age. As a result, approximately half of Japanese breast cancer patients at age 52 were premenopausal (Fig. 3). The data may aid the clinicians to decide whether to begin aromatase inhibitors for menopausal patients who are not menstruating after chemotherapy or tamoxifen. The distribution of breast cancer patients by age of onset is shown in Fig. 4. The bimodal distribution of onset in late 40 s and late 60 s is unique in Japanese patients and there has been a similar trend for years. We also analyzed the data on body mass index by age. As shown in Fig. 5, the body mass index of Japanese patients steadily increases after their late 40 s. Proper control of their own body weight is recommended, because obesity is known as one of risk factors for postmenopausal breast cancer.

Our data show the comparison of clinical and pathological classifications on tumor size and nodal status in 76,865 patients without preoperative systemic therapy and M1 disease (Table 2). Pathological T1 classification was similar in the number relative to that in clinical T1 classifications, while only 39.3% of the clinical Tis cases were diagnosed as Tis pathologically (Table 2a), suggesting clinical Tis may be overestimated. Thus, our data revealed that there were not a few differences between clinical and pathological Tis evaluations. Furthermore, of 68,872 clinical node-negative cases, 52,126 (75.5%) was node negative but 12.1% was node-positive pathologically, while of 7730 clinical node-positive cases, 6231 (80.6%) was node positive but 10.6% was node-negative pathologically (Table 2b). From this result, it is necessary to pay close attention to the selection of the surgical procedure.

The frequencies of lymph node metastasis by pathological tumor size and subtype in patients without neoadjuvant chemotherapy (NAC) are shown in Fig. 6. HER2-positive and triple negative breast cancer had high rates of lymph node metastasis compared to ER+ /HER2– disease. For example, approximately 15% of pT1c disease had lymph node metastasis, while more than 30% of T2 cases had positive lymph nodes. Treatment should be selected based on such essential information as it when considering NAC or surgery.

Finally, our data show the frequency of subtypes classified based on ER, PgR, and HER2 expression from immunohistochemical staining, which is fundamental data of the population of Japanese breast cancer patients (Fig. 7). There were differences in these biological characteristics between M0 and M1 disease. In M1 cases, there was increased ER negativity, PgR negativity, HER2 positivity, and nuclear grade 3 (Table 3). These factors should be considered first when evaluating biological features of individual breast cancer.

## Postscript

The data input to JBCS registry has varied over time. This registry also needs to be gradually taking in the opinions of clinicians and balancing it with what has not changed. At the same time when we register new cases, we need to analyze, discuss, publish, and progressively develop JBCS registry. We believe that this annual data report provides significant information to guide daily medical care for breast cancer patients.
